# Medication Safety Practice in Selected Saudi Hospitals: Alignment with the World Health Organization Global Medication Safety Challenge—An Exploratory Study

**DOI:** 10.3390/healthcare14121615

**Published:** 2026-06-08

**Authors:** Ghadah H. Alshehri, Layan S. Alaqil, Linah M. Alghamdi, Alaa A. Alsharif, Nada A. Alsaleh, Amani S. Alrossies, Aseel S. Abuzour, Asma M. Alshahrani, Amani A. Al Shaban, Douha F. Bannan

**Affiliations:** 1 Department of Pharmacy Practice, College of Pharmacy, Princess Nourah bint Abdulrahman University, Riyadh 11671, Saudi Arabia; aaalsharif@pnu.edu.sa (A.A.A.);; 2 College of Pharmacy, Princess Nourah bint Abdulrahman University, Riyadh 11671, Saudi Arabia; 3 Pharmaceutical Care Services, King Abdullah bin Abdulaziz University Hospital, Riyadh 11671, Saudi Arabia; 4 Faculty of Medicine and Health, School of Medicine, University of Leeds, Leeds LS2 9JT, UK; 5 Therapeutic Affairs Deputyship, Ministry of Health, Riyadh 12382, Saudi Arabia; 6 Pharmaceutical Care and Formulary Management Affairs, Pharmaceutical Care Standardization and Optimization, Eastern Health Cluster, Dammam 32253, Saudi Arabia; aaalshaban@moh.gov.sa; 7 Department of Pharmacy Practice, Faculty of Pharmacy, King Abdulaziz University, Jeddah 21589, Saudi Arabia

**Keywords:** medication safety challenge, medication safety, hospitals, Saudi Arabia

## Abstract

**Highlights:**

**What are the main findings?**

- This study offers a national snapshot of medication safety practices in selected Saudi hospitals according to the WHO Global Medication Safety Challenge framework, highlighting current areas of focus and identifying gaps, particularly in polypharmacy management and patient/public engagement.

**What are the implications of the main findings?**

- By identifying priority areas and gaps in medication safety practices, this study can guide future research, policymaking, and intervention design, to support safer medication use and facilitate the translation of global safety frameworks into local clinical practice.

**Abstract:**

**Background:** In response to the World Health Organization’s Global Medication Safety Challenge, many countries, including Saudi Arabia, have launched implementation initiatives. This study examined how Saudi hospitals have addressed the WHO Global Medication Safety Challenge. **Methods:** A cross-sectional survey was conducted between January and June 2025 among medication safety officers at secondary and tertiary care hospitals in Saudi Arabia. Participants reported interventions implemented to enhance medication safety during or before 2025 using a self-administered survey adapted from a previously published tool. Interventions were categorized according to the domains, subdomains, and priority areas of the WHO Medication Safety Challenge framework. Descriptive statistics summarized responses, and thematic analysis was used to categorize interventions. **Results:** Twenty-eight hospitals reported 162 interventions. Most respondents were medication safety officers (75%). The most frequently reported domains were medication systems and practices (41.3%), followed by healthcare professionals (29%), medicines (22.5%), and patients/public (6.7%). Nearly 60% of interventions addressed WHO priority areas, primarily high-risk situations (81.4%), followed by transitions of care (11.3%) and polypharmacy (7.2%). **Conclusions:** The study findings suggest that selected hospitals in Saudi Arabia have primarily focused on medication systems, healthcare professionals, and high-risk situations. Expanding initiatives to address polypharmacy and patient/public engagement may further strengthen medication safety efforts.

## 1. Introduction

Safe medication use is a priority for global health systems, and this is reflected in the World Health Organization (WHO), which highlights medication safety as a focus of its global patient safety initiatives [1]. Over the past few decades, the WHO has set agendas for countries to develop and implement interventions to improve healthcare delivery, reduce risk, and enhance patient outcomes. Accordingly, the WHO has launched three global patient safety challenges. The first two—Clean Care is Safer Care and Safe Surgery Saves Lives—aimed to raise global awareness of the risks associated with healthcare-associated infections and surgical procedures [2,3].

In 2017, the WHO introduced its third global Medication Safety Challenge (MSC), Medication Without Harm, which aimed to reduce the global prevalence of severe and preventable medication harm by 50% over five years [1]. As part of this challenge, the WHO called on countries to implement the challenge’s strategic framework, which includes four core domains—patients and the public, healthcare professionals, medicines, and systems and practices of medication—each of which is further divided into four subdomains (see Table 1). Moreover, the challenge identifies three priority areas: polypharmacy [4], high-risk situations [5], and transitions of care [6]. According to the WHO, these areas require early action by countries and key stakeholders to address and protect patients from medication-related harm.

In alignment with the WHO challenge, several countries undertook initiatives to examine the scale of preventable medication harm and to propose strategies for its reduction. In 2020, Australia released a national response to the WHO challenge in a report that evaluated relevant existing programs and refined them to better align with the Australian context [7]. Similarly, England’s Department of Health and Social Care commissioned an evidence-based review to examine the prevalence and severity of medication-related harm in hospitals. In this review, the number of medication errors that occurred during the medication treatment process in England was estimated at 200 million errors annually, 27% of which were potentially harmful. The report also indicated the cost of medication errors to the National Health System (NHS), which was estimated to be about £1.6 billion per year [8]. As part of this initiative, the Department of Health and Social Care established a Short Life Working Group to identify priority areas to address the challenges in England’s health care system, such as improved shared decision making, improved medication packaging and labeling, enhancement of electronic prescribing and administration systems and health care professionals’ encouragement of reporting and learning [9].

Saudi Arabia has demonstrated an interest in strengthening medication safety practices within hospitals [10]. This interest is connected to Saudi Vision 2030, which identifies enhancing the safety and quality of the healthcare system as one of its strategic pillars [11,12]. Accordingly, the Ministry of Health (MOH), through the General Administration of Pharmaceutical Care, maintains a national database for medication error reporting to generate learning opportunities aimed at enhancing medication safety in hospitals [13,14,15]. In addition, the Saudi Patient Safety Center was established to promote safe medication use in the Saudi healthcare system [16]. Moreover, the Saudi Central Board for Accreditation of Healthcare Institutions (CBAHI), the official body responsible for setting and monitoring safety and quality standards in healthcare facilities across Saudi Arabia, has developed standards to enhance medication safety within the healthcare system [17]. These standards require, for example, the presence of medication safety officers in accredited healthcare facilities to oversee and manage medication-related issues.

In terms of the studies that explored the extent to which countries have incorporated the WHO Medication Safety Challenge, a study by Garfield et al. examined how a sample of four English hospitals had incorporated the WHO challenge into their healthcare systems and provided a comprehensive overview of the interventions implemented related to the MSC as well as areas that require further attention [18]. However, to our knowledge, no study has systematically documented how Saudi hospitals (a country with a rapidly evolving healthcare system) have implemented and addressed the recommendations outlined in the WHO’s Medication Safety Challenge. Therefore, this exploratory study aims to assess whether, and how, selected hospitals in Saudi Arabia have responded to the “WHO Global Patient Safety Challenge strategic framework”.

## 2. Materials and Methods

### 2.1. Study Design and Setting

A cross-sectional, survey-based study was conducted with medication safety officers. In case a medication safety officer was unavailable, whether due to leave, holidays, or workload, other healthcare professionals familiar with medication safety practices completed the survey. A purposive sampling strategy was employed to identify and recruit participants. The sample framework utilizes a medication safety officer and equivalent roles from each hospital that were accredited by the CBAHI. Participants were identified through the research team’s professional networks. LinkedIn was additionally used to identify and reach eligible medication safety officers when their professional contact details were not available. The medication safety officers were invited to report interventions implemented to improve medication safety, specifically those introduced during or prior to 2025. Subsequently, the research team categorized the reported interventions according to the domains, subdomains, and priority areas identified in the MSC [1]. The categorization was independently conducted and coded by two researchers with expertise in medication safety, and any discrepancies were resolved by discussion and consensus. The hospitals involved were all located in Saudi Arabia and included both secondary and tertiary care facilities. The study aimed to document and categorize medication safety interventions that are in accordance with the WHO MSC framework, and to exploratorily assess alignment.

### 2.2. Data Collection

An English-language survey was developed and comprised two sections. In the first section, participants were asked to provide general demographic and employment information, including their job title, hospital type (secondary or tertiary), and hospital region (i.e., Eastern, Western, Southern, or Central regions). The second section included a single open-ended question that asked participants to describe an intervention that had been or was currently being implemented to enhance medication safety practices in their organization (see Supplementary Materials). The final survey questions were pilot-tested with ten participants, and revisions were made accordingly, including rewards to enhance clarity and addition of an overview of the WHO’s MSC to support participants in completing the survey. The survey link was distributed using REDCap^®^ (Research Electronic Data Capture) platform [19] to eligible participants via their professional contact details, along with a brief description of the study’s purpose. To ensure confidentiality and anonymity, no personally identifiable information, such as participants’ and hospital names, was collected. Participation was voluntary, and informed consent was obtained before completing the survey.

The inclusion criteria included (1) secondary and tertiary care hospitals in Saudi Arabia with established medication safety programs, (2) institutional familiarity with medication safety practices, (3) currently employed as a medication safety officer, and (4) willingness to participate in the study. Exclusion criteria included: (1) primary care settings, (2) incomplete responses, and (3) responses that are not medication interventions.

### 2.3. Statistical Analysis

Descriptive analysis was used to summarize the characteristics of the participating hospitals and respondents. Categorical variables, such as hospital type (i.e., secondary or tertiary), region (i.e., Eastern, Western, Central, Southern), and professional role (e.g., medication safety officer, pharmacist, quality specialist), were reported as frequencies and percentages. Open-ended responses describing medication safety interventions were reviewed qualitatively and categorized thematically based on the domains, subdomains, and three priority areas identified in the Medication Safety Challenge’s framework. The frequency of interventions reported within each domain and priority area was then summarized using descriptive statistics (counts and percentages). All quantitative analyses were conducted using Microsoft Excel^®^, June 2025.

### 2.4. Ethical Considerations

Institutional Review Board Statement: This study was approved by King Abdullah bin Abdulaziz University Hospital Institutional Review Board on 22 February 2024 (IRB Log Number: 24-0027). An Informed Consent Statement was obtained electronically from all participants before filling out the survey.

## 3. Results

### 3.1. Overview

**Figure 1:**
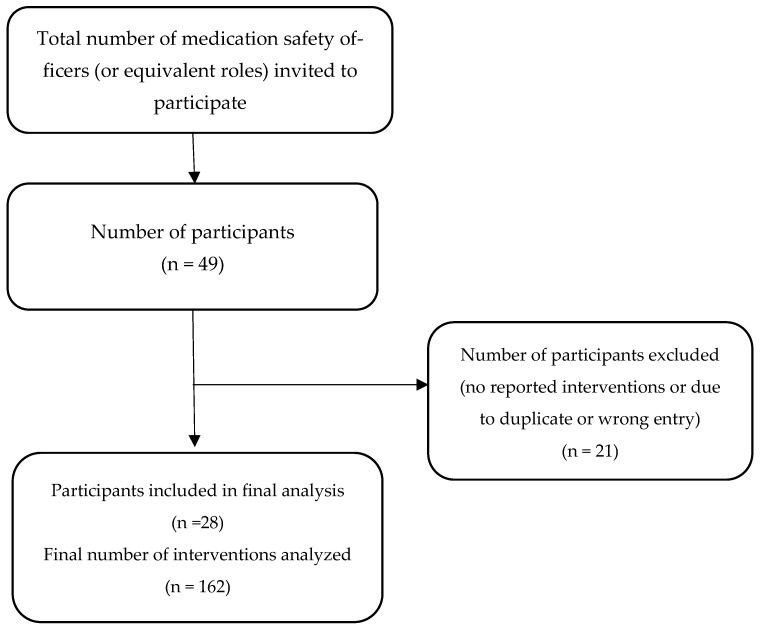
Flow chart of the participants’ response screening.

A total of 54 (out of 516 CBAHI-accredited hospitals) [20] medication safety officers or equivalent eligible respondents, each representing a different hospital, were invited; 49 participants completed, yielding a 90.7% response rate. Twenty-one participants (n = 21) were excluded due to duplicate or incorrect entries (e.g., descriptions of medication errors rather than interventions). The final dataset included 28 participants (57.1%), with a total of 162 interventions reported (see Figure 1). Among the participants, 21 were employed as medication safety officers (n = 21). When the medication safety officer was unavailable, other healthcare professionals familiar with medication safety practices completed the survey, including one chair of a hospital-based medication safety center, three heads of pharmacy, one clinical pharmacy services manager, one deputy director of pharmacy services, and one drug information supervisor.

Across all regions, a total of 28 hospitals participated in the study. The highest number of responses was from the Western region, which included 12 hospitals (42.9%), of which 8 were secondary care hospitals, and 4 were tertiary care hospitals. The Eastern region followed with nine hospitals (32.1%), all of which were tertiary care hospitals. In the Central region, four hospitals (14.3%) participated, including one secondary care hospital and three tertiary care hospitals. Lastly, the Northern region accounted for three hospitals (10.7%), all of which were secondary care hospitals.

### 3.2. Medication Safety Challenge Framework and Priorities

From a total of 162 interventions submitted, system and practice in medication (n = 67/162; 41.3%) emerged as the most frequently reported. These interventions primarily focused on enhancing medication safety practices throughout the medication-use process, beginning with prescribing and extending through the monitoring stage. Accordingly, the most cited subdomain was prescribing, preparation, and dispensing (n = 47/67; 70.1%), which included initiatives such as implementing smart pharmacies and robotics technologies for medication preparation and dispensing. This was followed by administration and patient monitoring (n = 7/67; 10.4%), including interventions such as implementing smart syringe infusion pumps to control IV medication administration.

The second most frequently reported domain was healthcare professionals (n = 47/162; 29%. Most interventions in this category focused on education and training (n = 24/47; 51%, such as hosting workshops on IV medication preparation, High-Alert Medications (HAMs), and medication-dose calculations. The second most prominent subdomain was incident reporting and learning (n = 13/47; 27.7%), which included implementing electronic medication error reporting systems with monthly analyses and shared feedback (Table 2).

The third most reported domain was medicines (n = 36/162; 22.2%). The most frequently reported subdomain was product quality and safety (n = 19/36; 52.8%), including interventions aimed at enhancing medication safety and quality protocols. Examples included implementing dosing and pharmacokinetic monitoring protocols for agents such as vancomycin, gentamicin, and amikacin. The second most common subdomain was logistics, storage, and disposal (n = 10/36; 27.8%), which included interventions such as optimizing medication storage based on risk categorization and ensuring barcoding and expiry labeling.

Patients and the public (n = 11/162; 6.8%) were the least frequently reported domain. Two subdomains were reported: patient engagement (n = 9/11; 81.8%) and public awareness and medication literacy (n = 2/11; 18.2%). Patient engagement interventions included establishing virtual counseling clinics for polypharmacy patients to support complex medication regimens, including anticoagulants, neurology, oncology, and transplant therapies. Public awareness interventions included launching World Patient Safety campaigns to promote safe medication use (Table 3).

In terms of the MSC’s priority areas, a total of 97 (59.8%) interventions directly targeted all three priority areas. High-risk priority areas (n = 79/97; 81.4%) were the most frequently reported and included the implementation of a High-Alert Medication (HAM) double-check system that includes a mandatory independent double-check procedure for HAMs using checklist-based verification and a two-person verification policy.

Transition of care (n = 11/97; 11.3%) represented the next largest proportion of reported interventions and included, for example, the implementation of electronic medication reconciliation using a robust platform that is easily accessible to pharmacists and physicians and enables them to verify patient medication lists across different points of care to prevent medication discrepancies. Finally, the least-reported priority area was polypharmacy (n = 7/97; 7.2%), the use of multiple medications by a patient. One example of an intervention in this area is the Deprescribing Initiative in Geriatric Care project, which involves the systematic review of elderly patients’ medications to discontinue unnecessary or potentially harmful drugs. Table 4 summarizes the numbers and examples of reported interventions related to MSC priority areas.

## 4. Discussion

Most interventions reported by participating hospitals focused on the systems and practices of medication management, including prescribing, dispensing administration, and monitoring, followed by the medicine domains, especially product safety and quality. The patients and the public domain were the least represented, and interventions in this domain fell into only two subdomains: medication literacy and patient engagement and public awareness. Additionally, we found that most of the interventions addressed the challenge’s priority areas at different levels, with high-risk situations and transition of care being the most frequent priority areas reported. Overall, the findings indicate that while substantial progress has been made in strengthening medication management systems, there are still opportunities to broaden efforts focused on patient engagement and public involvement, moving toward a fully integrated framework for medication safety.

A comparison with international experiences reveals similarities in the implementation of the WHO’s MSC. Much like our findings, most of the interventions reported by four National Health Service (NHS) hospitals in England addressed systems and practices of medication management as well as the healthcare professional domains as the most frequently reported. Similarly, in terms of the challenge priority areas, both studies identified high-risk situations as the most frequently targeted, followed by the transition of care and polypharmacy [18]. Notably, the England study adopted an interview and focus group approach, which provides deeper insight but may be limited in geographical coverage, while the present study was based on survey data that enables broader geographical coverage. Harmonizing these two methods strengthens confidence in pattern generation, and the similarity observed in the domains and priority areas supports the consistency of the findings across both studies. Concerning Australia, a 2024 report responded to the challenge, specifically the three priority areas, and was supported by 12 defined action plans, along with associated performance metrics. The Australian report emphasized the implementation of digital infrastructure and active consumer involvement [21]. Collectively, these results point to a shared global alignment on the WHO MSC, but with varying levels of implementation.

While this study did not directly measure implementation of policy, it highlighted interventions focused on enhancing systems and practices of medication management through digital technologies that align with the Saudi Vision 2030 objectives, specifically those listed in the Health Sector Transformation Program, which emphasizes digital health as a core component of healthcare improvement [12]. Several hospitals reported the use of digital tools, such as computerized physician order entry (CPOE) systems, automated dispensing cabinets, barcode scanning technology, electronic medical records (EMRs), and smart pharmacy solutions that incorporate robotics technology [12]. Such advancements illustrate the ongoing national commitment to integrating digital tools in support of safe and more efficient medication practices. According to a national survey carried out by Alsultan et al. (2012), 34.5% of Saudi hospitals have adopted CPOE systems and clinical decision support systems (CDSSs) [22]. These figures provide a historical perspective on the adoption of technology in Saudi hospitals. Moreover, 21% of Saudi hospitals regularly employ barcode systems and automated dispensing cabinets, and approximately one-third of hospitals have implemented electronic medication administration records (eMARs). Although only 7.4% are using barcode-assisted medication administration, 12% have adopted smart infusion pump technology [23]. Although these findings provide valuable insight into the extent of digital tool implementation in Saudi hospitals, future studies are warranted to showcase the ongoing advancement and their roles in enhancing medication safety practice.

Although the patient and public domain was the least addressed, several national policy initiatives have been launched to support it. In 2023, for example, the Saudi Food and Drug Authority released guidance on patient engagement that encourages the active involvement of patients in regulatory decision-making, clarifies their roles, and facilitates discussion around the safety and the efficacy of medical products [24]. Another key initiative is the implementation of EMRs, which enable patients to access their health-related information and medication treatment plans while also facilitating communication with healthcare providers [25]. Based on previous EMR research, this initiative is expected to improve healthcare quality, enhance service delivery, and streamline workflows for healthcare professionals [26,27,28]. Moreover, in 2019, the Saudi Patient Safety Center established a patient and family advisory council that aims to promote patient engagement and act as a crucial link between patients and healthcare providers to ensure that patients’ perspectives are integrated into healthcare practices [16]. Future studies should endeavor to better understand this discrepancy between national and hospital priorities, with the goal of identifying approaches to enhance patient and public engagement at all institutional levels.

At present, Saudi hospitals appear to prioritize the high-risk situation priority area, which is the one most associated with medication harm due to errors [29]. This focus is attributable in part to the CBAHI standard, which requires hospitals to have systems in place that manage High-Alert Medication (HAM) and Look-Alike Sound-Alike (LASA) safety [17]. Alomi et al. evaluated Saudi hospital pharmacists’ high-risk medication and HAM practices and found that pharmacists generally reported positive implementation of these practices [30]. This strong focus on high-risk medications reflects a standards-driven strategy and focuses resources on interventions with the highest potential impact.

This study of medication safety practices in selected hospitals in Saudi Arabia provides a snapshot of implemented interventions that align with the WHO’s MSC Medication Without Harm. The results reflect current national efforts aimed at improving patient safety and enhancing the quality of healthcare practices. Moreover, our findings contribute important insights into the adoption of a specific international safety framework in Saudi Arabia to the global body of knowledge on implementing patient safety strategies [18,21].

Nevertheless, this study has several limitations. While these findings provide preliminary insight, they should be interpreted with caution and treated as hypothesis-generating rather than used to generalize to a national estimate, as they rely on self-reported data. A particular concern with self-reporting is that newly employed respondents may have been unaware of interventions implemented before their hiring date. The General Authority for Statistics’ Healthcare Establishment and Workforce Statistics in 2025 report that Saudi Arabia has about 516 hospitals nationwide [20]. In our study, a sample size of 28 hospitals represents approximately 5.4% of Saudi Arabian hospitals, along with the predominance of responses from the Western region despite nationwide survey distribution, and the use of purpose sampling may collectively limit the national generalizability. In addition, the absence of a stratified, institution-based sampling approach and reliance on recruitment through professional networks and networking platforms rather than a systematic sampling frame may introduce selection bias. Non-medication safety officers (such as clinical pharmacy managers and heads of pharmacy) completed the survey in the absence of medication safety officers; while they are familiar with medication safety practices, their roles and perspectives may differ, which could potentially influence the study findings. The study did not objectively measure the actual level of intervention implementation, which represents an area for future research. Finally, while inconsistencies in categorizing interventions could be a potential limitation, this risk was mitigated through independent categorization and validation by two members of the research team.

Future studies should include larger, more representative samples from various regions within Saudi Arabia to enhance the generalizability of the study findings. Further research needs to investigate how patients and the public are involved in efforts towards ensuring medication safety, especially in cases of polypharmacy and high-risk situations. Additionally, longitudinal research is essential for examining the trends of medication safety practices in Saudi Arabia over time, in line with the Vision 2030 healthcare development plan.

## 5. Conclusions

The study findings show that selected hospitals in Saudi Arabia have made efforts to implement the WHO MSC strategic framework, especially in medication safety practices and healthcare professional domains with respect to high-risk situations. However, these findings should be cautiously interpreted because of the exploratory nature and the limited number of hospitals in the study and should not be generalized to all hospitals in Saudi Arabia. Further research is needed to validate these findings and inform policy makers to target medication safety initiatives in Saudi Arabia.

## Figures and Tables

**Table 1:** Strategic framework of the global patient safety challenge and priority areas.

Domain (Key Action Area)	Subdomains
Patients and the Public	(1) Public awareness and medication literacy. (2) Patient engagement. (3) Reporting by patients. (4) Involvement of patient organizations
Health Care Professionals	(1) Education and training. (2) Communication and teamwork. (3) Capability at point of care. (4) Incident reporting and learning
Medicines	(1) Product quality and safety. (2) Naming, labeling and packaging. (3) Logistics, storage and disposal
Systems and Practices of Medication	(1) Leadership and governance. (2) Prescribing, preparation and dispensing. (3) Administration and patient monitoring. (4) Monitoring and evaluation
Priority Areas	(1) High-risk situation. (2) Polypharmacy. (3) Transition of care

**Table 2:** Distribution of medication safety interventions by WHO MSC domain and subdomain.

Domain	Subdomain	Interventions, n (%)
Medication management systems and practices (n = 67)	Prescribing, preparation, dispensing	47 (70.2)
	Administration and patient monitoring	13 (19.4)
	Monitoring and evaluation	7 (10.4)
Health care professionals (n = 47)	Education and training	24 (51.0)
	Incident reporting and learning	13 (27.7)
	Communication and teamwork	2 (4.3)
	Capability at the point of care	8 (17.0)
Medicines (n = 37)	Product quality and safety	19 (51.4)
	Logistics, storage and disposal	10 (27.0)
	Right product at point of care	3 (8.1)
	Naming, labeling and packaging	5 (13.5)
Patients and the public (n = 11)	Patient engagement	9 (81.8)
	Public awareness and medication literacy	2 (18.2)

**Table 3:** Representative examples of interventions by domain.

Domain	Representative Intervention Example
Medication systems	Optimizing preoperative prophylaxis timing (prescribing to administration within 60 min)
	Closed-loop medication barcoding at administration
	Darbepoetin monitoring protocol following near-miss trend
Health care professionals	Annual sterile compounding competency training and certification
	Daily medication safety huddles in high-risk units
	Pharmacist participation in multidisciplinary rounds
Medicines	Formulary removal of iron salts after adverse event review
	Shifting IV preparation to post-rounds to reduce wastage by 44%
	High-Alert Medication independent double-check system
Patients and the public	Multilingual patient/family medication education at discharge
	In-hospital World Patient Safety Day awareness campaign

**Table 4:** Interventions targeting the WHO Medication Safety Challenge priority areas.

Priority Area	Interventions Identified (n = 97)	Examples of Interventions
High-risk situations	79	Standardized intravenous norepinephrine infusion for shock patients as a first-line treatment at a step-down unit (starting dose of 0.01 mcg/kg/min up to 0.04 mcg/kg/min) to replace the previous dopamine infusion.
Transitions of care	11	Implementation of a medication reconciliation system is mandatory at admission, during care transitions, and at discharge. The system includes retrieving the patient’s medication history, providing guidance for conducting patient interviews, and requiring review and acknowledgment by a pharmacist or clinical pharmacist.
Polypharmacy	7	Implementation of an inpatient medication de-prescribing protocol for antihyperglycemic agents, applied through a multidisciplinary approach and guided by validated tools such as the Beers Criteria and the STOPP/START guidelines.

## Data Availability

The original contributions presented in this study are included in the article/Supplementary Materials. Further inquiries can be directed to the corresponding author.

## Supplementary Materials

The following supporting information can be downloaded at: https://www.mdpi.com/article/10.3390/healthcare14121615/s1.
