# Supplementary material for: Medication Safety Practice in Selected Saudi Hospitals: Alignment with the World Health Organization Global Medication Safety Challenge—An Exploratory Study
Source: Healthcare (Basel). 2026 Jun 8;14(12):1615. doi: 10.3390/healthcare14121615 (PMC13299394; doi:10.3390/healthcare14121615)
Supplement: Supplementary file 1 [file healthcare-14-01615-s001.zip › healthcare-4342201-supplementary.pdf]

Exploring How Hospitals in Saudi Arabia Are Addressing the WHO's Medication Safety Challenge

Dear Participant,

This survey targets Pharmacy Managers, Medication Safety Officers, and healthcare professionals involved in enhancing medication safety practices in Saudi hospitals. We seek your input on interventions implemented at your institution following the launch of the World Health Organization’s (WHO) Third Global Patient Safety Challenge, “Medication Without Harm,” which encourages strategies to reduce medication errors.

□□ To learn more about the WHO initiative: https://www.who.int/initiatives/medication-without-harm

We are inviting over 100 hospitals across Saudi Arabia to participate. The survey takes approximately 15–20 minutes, and all responses will remain strictly confidential.

You’ll be asked to describe medication safety interventions introduced at your institution since 2017. Please include the name of each intervention and a detailed description of its key features and implementation process. The more detailed your input, the better we can classify it according to WHO’s domains and priorities.

This study has received ethical approval from the Institutional Review Board (IRB). Data collected via the RedCap system will be securely stored and destroyed after five years. No individual hospitals will be identified, and no data will be shared with third parties.

For any questions or assistance, please contact Dr. Ghadah H. Alshehri at ghalshehri@pnu.edu.sa.

Thank you for your time and valuable contribution.

Do you consent to participate on this online questionnaire ?
- ◯ Yes
- ◯ No

Please write your email ______________________________

What is your job title ?
- ◯ Medication safety officer
- ◯ Head of the pharmacy
- ◯ Senior pharmacist
- ◯ Other

Please write, If you choose (Other) ______________________________

Does your hospital have medication safety officer?
- ◯ Yes
- ◯ No

How many medication safety officer do you have ? ______________________________

If no, who is responsible for addressing the medication safety problems ? ______________________________

Region of the hospital
- ◯ Central region
- ◯ Western region
- ◯ Eastern region
- ◯ Southern region
- ◯ Northern region

Please write the name of your city ______________________________

Type of the hospital
- ◯ Secondary
- ◯ Tertiary

How many interventions have been implemented to target the four domains of the Challenge?
* In the next sections, you can list up to 15 interventions implemented at your institution. If there are none, simply choose 'None,' and the survey will close automatically.

____
((Add number MAX 15))

Do you have a first intervention ?

◯ Yes
◯ No

Please provide the name of the intervention and describe it in as much detail as possible, including its features and implementation steps. The more detailed the description, the better we will be able to classify the intervention according to the World Health Organization's domains and priority classifications.

____

Do you have a 2 intervention ?

◯ Yes
◯ No

Please provide the name of the intervention and describe it in as much detail as possible, including its features and implementation steps.

____

Do you have a 3 intervention ?

◯ Yes
◯ No

Please provide the name of the intervention and describe it in as much detail as possible, including its features and implementation steps.

____

Do you have a 4 intervention ?

◯ Yes
◯ No

Please provide the name of the intervention and describe it in as much detail as possible, including its features and implementation steps.

____

Do you have a 5 intervention ?

◯ Yes
◯ No

Please provide the name of the intervention and include a brief description

____

Do you have a 6 intervention ?

◯ Yes
◯ No

Please provide the name of the intervention and describe it in as much detail as possible, including its features and implementation steps.

____

Do you have a 7 intervention ?

◯ Yes
◯ No

Please provide the name of the intervention and describe it in as much detail as possible, including its features and implementation steps.

____

Do you have an 8 second intervention ?

◯ Yes
◯ No

Please provide the name of the intervention and describe it in as much detail as possible, including its features and implementation steps. ______________________

Do you have a 9 intervention ?
◯ Yes
◯ No

Please provide the name of the intervention and describe it in as much detail as possible, including its features and implementation steps. ______________________

Do you have a 10 second intervention ?
◯ Yes
◯ No

Please provide the name of the intervention and describe it in as much detail as possible, including its features and implementation steps. ______________________

Do you have an 11 intervention ?
◯ Yes
◯ No

Please provide the name of the intervention and describe it in as much detail as possible, including its features and implementation steps. ______________________

Do you have an 12 intervention ?
◯ Yes
◯ No

Please provide the name of the intervention and describe it in as much detail as possible, including its features and implementation steps. ______________________

Do you have a 13 intervention ?
◯ Yes
◯ No

Please provide the name of the intervention and describe it in as much detail as possible, including its features and implementation steps. ______________________

Do you have a 14 intervention ?
◯ Yes
◯ No

Please provide the name of the intervention and describe it in as much detail as possible, including its features and implementation steps. ______________________

Do you have a 15 intervention ?
◯ Yes
◯ No

Please provide the name of the intervention and describe it in as much detail as possible, including its features and implementation steps. ______________________
